# Effects of Inositol-Enhanced Bonded Arginine Silicate Ingestion on Cognitive and Executive Function in Gamers

**DOI:** 10.3390/nu13113758

**Published:** 2021-10-24

**Authors:** Ryan Sowinski, Drew Gonzalez, Dante Xing, Choongsung Yoo, Victoria Jenkins, Kay Nottingham, Broderick Dickerson, Megan Humphries, Megan Leonard, Joungbo Ko, Mark Faries, Wesley Kephart, Christopher J. Rasmussen, Richard B. Kreider

**Affiliations:** 1 Exercise & Sport Nutrition Laboratory, Human Clinical Research Facility, Department of Health & Kinesiology, Texas A&M University, College Station, TX 77843, USA; ryansowinski6@gmail.com (R.S.); dg18@tamu.edu (D.G.); dantexing@tamu.edu (D.X.); choongsungyoo@tamu.edu (C.Y.); victoria.jenkins@tamu.edu (V.J.); kvnottingham@tamu.edu (K.N.); dickersobl5@email.tamu.edu (B.D.); mhumphries@tamu.edu (M.H.); meganleonard10@tamu.edu (M.L.); joungboko10@tamu.edu (J.K.); Mark.Faries@ag.tamu.edu (M.F.); crasmussen@tamu.edu (C.J.R.); 2 Texas A&M AgriLife Extension, Texas A&M University, College Station, TX 77843, USA; 3 Department of Kinesiology, University of Wisconsin–Whitewater, Whitewater, WI 53190, USA; kephartw@uww.edu

**Keywords:** esports, cognition, memory, reaction time, attention, vigilance, psychomotor skill

## Abstract

Inositol stabilized arginine silicate (ASI) ingestion has been reported to increase nitric oxide levels while inositol (I) has been reported to enhance neurotransmission. The current study examined whether acute ASI + I (Inositol-enhanced bonded arginine silicate) ingestion affects cognitive function in e-sport gamers. In a double blind, randomized, placebo controlled, and crossover trial, 26 healthy male (n = 18) and female (n = 8) experienced gamers (23 ± 5 years, 171 ± 11 cm, 71.1 ± 14 kg, 20.7 ± 3.5 kg/m^2^) were randomly assigned to consume 1600 mg of ASI + I (nooLVL^®^, Nutrition 21) or 1600 mg of a maltodextrin placebo (PLA). Prior to testing, participants recorded their diet, refrained from consuming atypical amounts of stimulants and foods high in arginine and nitrates, and fasted for 8 h. During testing sessions, participants completed stimulant sensitivity questionnaires and performed cognitive function tests (i.e., Berg-Wisconsin Card Sorting task test, Go/No-Go test, Sternberg Task Test, Psychomotor Vigilance Task Test, Cambridge Brain Sciences Reasoning and Concentration test) and a light reaction test. Participants then ingested treatments in a randomized manner. Fifteen minutes following ingestion, participants repeated tests (Pre-Game). Participants then played their favorite video game for 1-h and repeated the battery of tests (Post-Game). Participants observed a 7–14-day washout period and then replicated the study with the alternative treatment. Data were analyzed by General Linear Model (GLM) univariate analyses with repeated measures using weight as a covariate, paired *t*-tests (not adjusted to weight), and mean changes from baseline with 95% Confidence Intervals (CI). Pairwise comparison revealed that there was a significant improvement in Sternberg Mean Present Reaction Time (ASI + I vs. PLA; *p* < 0.05). In Post-Game assessments, 4-letter Absent Reaction Time (*p* < 0.05), 6-letter Present Reaction Time (*p* < 0.01), 6-letter Absent Reaction Time (*p* < 0.01), Mean Present Reaction Time (*p* < 0.02), and Mean Absent Reaction Time (*p* < 0.03) were improved with ASI + I vs. PLA. There was a non-significant trend in Pre-Game Sternberg 4-letter Present Reaction time in ASI + I vs. PLA (*p* < 0.07). ASI + I ingestion better maintained changes in Go/No-Go Mean Accuracy and Reaction Time, Psychomotor Vigilance Task Reaction Time, and Cambridge Post-Game Visio-spatial Processing and Planning. Results provide evidence that ASI + I ingestion prior to playing video games may enhance some measures of short-term and working memory, reaction time, reasoning, and concentration in experienced gamers.

## 1. Introduction

Gaming or “e-sports” has become a very popular activity particularly among younger individuals. It requires quick reactions, executive function, memory, and fine motor skill [1]. In e-sport competitions and tournaments, participants often play for hours per session over a series of days. Thus, the ability to maintain cognitive and executive function, concentration, and fine motor skill is paramount. For this reason, gamers often consume caffeinated energy drinks to help them stay alert and delay fatigue. However, excessive intake of caffeine may promote nervousness and/or interfere with fine motor skills [2] and/or have no effect on e-sport performance [3]. Therefore, there has been interest in identifying nootropic alternatives to caffeine. 

Inositol stabilized arginine silicate has been reported to increase blood levels of arginine and nitric oxide levels [4]. Nitric oxide (NO) promotes vasodilation in peripheral tissues as well as the brain [5,6,7]. Maintenance of adequate blood flow, oxygen availability, and delivery of nutrients to the brain have been suggest as important factors in maintenance of cognitive function and brain health as we age [6]. In this regard, increasing nitric oxide has been reported to enhance cognitive function and learning through several mechanisms including augmenting excitability of potassium channels, thereby mediating calcium-dependent activation of neuronal nitric oxide via nitrosylation of *N*-methyl-*D*-aspartic acid (NMDA) and regulating pathways involved in synaptic transmission [5,6]. Conversely, endothelial dysfunction, reductions in blood flow and oxygen delivery to the brain and compromised NO activity have been implicated in cognitive impairment [7]. In healthy individuals, increasing NO levels may enhance cerebral blood flow and oxygen availability in the brain, and thereby enhance cognitive function. Additionally, inositol has been reported to enhance neurotransmission [8] and memory [8,9,10]. Theoretically, co-ingesting ASI and inositol may affect cognitive function and/or memory in individuals who need to maintain focus, attention, and/or neurocognitive performance such as e-sport gamers. 

Two studies have evaluated the effects of ingesting ASI [11] and ASI + I [12] on cognitive function. In the first study, ASI supplementation (1500 mg/day for 3- days and 14- days) significantly improved the ability to perform complex cognitive tests requiring mental flexibility, processing speed and executive functioning. In the second study [12], adding 100 mg of inositol to the ASI significantly improved cognitive function and accuracy in gamers after playing video games for one hour. This purpose of this study was to expand on this work and determine whether ASI + I supplementation affects cognitive and executive function, and more specifically reaction time and working memory, in experienced gamers prior to and/or following a 1-h gaming challenge. Based on prior research, we hypothesized that ASI + I would promote improvements in cognitive and executive function in gamers. 

## 2. Methods

### 2.1. Experimental Design

This study was conducted in a university setting as a double blind, placebo controlled, and crossover trial. Nutritional supplementation served as the independent variable. Primary outcomes included assessment of cognitive and executive function and light tracking reaction time performance. Secondary outcomes included assessment of stimulant sensitivity and side effects assessment. 

### 2.2. Participants

This study was conducted with approval from the university’s Institutional Review Board (IRB2020-0181) in compliance with the Declaration of Helsinki standards for ethical principles regarding human participant research. This study was registered with clinicatrials.gov (NCT04828278). Healthy experienced gamers between 18–40 years of age with a body mass index (BMI) between 18–34.9 kg/m^2^ and no recent history of ingestion (<2 week) of dietary supplements which affect cognitive function were recruited to participate in the study. Participants were required to have a self-reported history of gaming 5 h/week. or more within the past 6 months and be willing to supply their own operator-oriented action or strategy video game they had played at least 21 times in the last 3 months, with the gaming platform and any accessories needed for play. Those who expressed interest were screened by phone to determine initial eligibility. Individuals meeting screening criteria were invited to attend a familiarization session where they reviewed the study design and testing procedures, signed informed consent statements, completed personal medical histories, and had a physical examination by a research assistant to assess qualifications. Participants were excluded from the study if they: (1) had a medical condition requiring physician prescribed medications (birth control was allowed); (2) a history of cognitive dysfunction, asthma, cirrhosis of the liver, guanidinoacetate methyltransferase deficiency, herpes, hypotension, a recent heart attack (≤1 year.), kidney disease; (3) scheduled surgery during the study; (4) gastrectomy; (5) bipolar disorder; (6) general allergies or allergies to maltodextrin, aspirin or tartrazine products; or, (7) was pregnant, breastfeeding, or planned to become pregnant within 4–5 weeks of starting study. 

**Figure 1:**
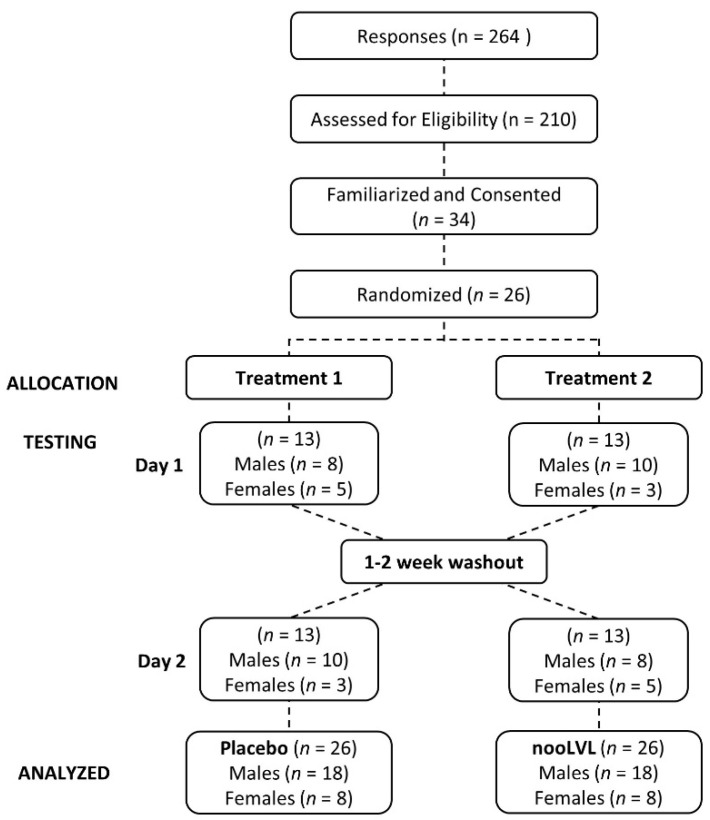
Consolidated Standards of Reporting Trials (CONSORT) diagram.

Figure 1 presents a Consolidated Standards of Reporting Trials (CONSORT) diagram showing that a total of 264 individuals responded to study advertisements, 210 were assessed for eligibility, 32 completed a familiarization session, 26 were randomized into treatments, and 26 participants completed the trial. Treatments are shown divided into testing days and show the number of and sex of participants (n) in each treatment on a given day of testing.

### 2.3. Testing Protocol

**Figure 2:**
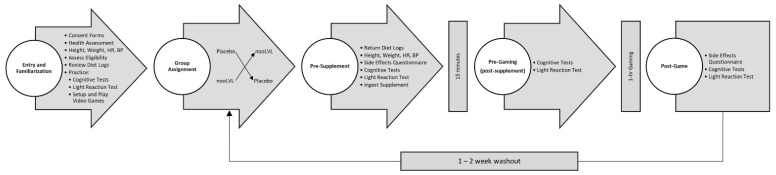
Overview of study protocol.

Figure 2 provides an overview of the experiment protocol. Participants attended a familiarization session and two experimental testing sessions during the study. During the familiarization session, participants were informed about the study protocol, signed informed consent statements, and underwent health screening that included measurement of height, weight, heart rate, and blood pressure. Participants then practiced each cognitive function tests and the light tracking test three times to familiarize them with the testing protocol and set-up, and practiced playing the video game in the testing settings. Prior to each testing session, participants recorded food and fluid intake for 4-days prior to their testing session and replicated food and fluid intake for 4-days prior to their second testing session. This included maintaining normal stimulant intake (caffeine < 200 mg/d) and refraining from any other stimulants, energy drinks, and foods high in arginine (e.g., beets, beet root juice, garlic, dark chocolate, green leafy vegetables such as spinach, arugula, kale, and cabbage) for 72-h prior to each testing session. Participants also fasted for 8–12 h prior to each testing session. Testing sessions lasted about 3–4 h and included setting up the gaming station and performing cognitive function and light reaction (Pre-Supplement); ingesting the assigned treatment, waiting 15-min, and performing the cognitive function and light reaction tests (Pre-Game); competitively gaming for 1-h; and, then performing the cognitive function and performance tests (Post-Game). After the first testing session, participants observed a 7 to 14-day washout period and then replicated their 4-day diet with the dietary restrictions noted above before repeating the experiment with the alternate supplement treatment.

### 2.4. Supplementation Protocol

Supplements were administered in a double blind, crossover, and randomized manner in generic stick pack tubes labeled A or B. One stick pack contained 1500 mg of arginine silicate, 100 mg of inositol, citric acid, natural flavor, sucralose, acesulfame potassium, silicone dioxide, and Red 40 (ASI + I, nooLVL^®^, Nutrition 21, Purchase, NY, USA). The placebo stick packs contained 3 g of tapioca maltodextrin, citric acid, natural flavor, sucralose, acesulfame potassium, silicone dioxide, and Red 40. Both supplements were packaged for double blind administration by Creative Concepts (Pittsburg, CA, USA). The contents and purity of each supplement was verified independently by Covance Eurofins Food Chemistry Testing (Madison, WI, USA). The powder supplements were mixed in 8oz of water with a flavored powder stick pack (CrystaLite^®^, Kraft Foods, Glenview, IL, USA) in a black shaker bottle (BlenderBottle^®^, Lehi, UT, USA). Supplements were ingested after completion of Pre-Supplement assessments. Participants observed a 7–14-day washout period between experimental sessions and then repeated the experiment while consuming the alternate treatment. 

## 3. Procedures

### 3.1. Dietary Assessment

Diet intake was assessed via a self-recorded account of all food and energy containing beverages over a 4-day period (3 weekdays and 1 weekend day) prior to each visit using the 2021 MyFitnessPal Calorie Counter smartphone app (MyFitnessPal, Inc., Baltimore, MD, USA) or written food logs. Food records were entered by study researchers, verified for consistency by one individual, and analyzed using the Food Processor Nutrition Analysis Software, Version 11.4.412 (ESHA Nutrition Research, Salem, OR, USA) [13]. 

### 3.2. Demographics

Height and weight were measured on a Health-O-Meter Professional 500 KL (Pelstar LLC, Alsip, IL, USA) self-calibrating digital scale (±0.02 kg). Resting hemodynamic measures were obtained in a seated position following approximately 6-min of rest. Heart rate was assessed via the radial artery, while blood pressure was measured by oscillation of the brachial artery using mercurial sphygmomanometer using standard procedures [14].

### 3.3. PEBL Cognitive Function Assessment

The Psychology Experiment Building Language (PEBL) software (Version 2.1, http://pebl.sourceforge.net, accessed on 7 April 2019) was used to administer the cognitive function test battery [15,16]. The PEBL test included the Berg-Wisconsin card sorting task test (BCST). In this test, visual stimuli (in the form of cards) are presented with instructions to sort the cards by matching colors and/or designs [15,16]. The test assesses reaction time and accuracy in measuring long thought, reasoning, learning, executive control, attention shifting by assessing inability to shift set (i.e., display flexibility in the face of changing schedules of reinforcement), and impulsiveness [17,18]. Test–retest reliability of correct responses during the familiarization session revealed a coefficient of variation (C_V_) of 4.9% and a standard error of mean expressed as a percent of the mean (SEM%) of 0.7%. The Go/No-Go test (GNG) was then administered. This test assesses sustained attention and response control through reaction time and accuracy of responding to visual stimuli (i.e., seeing P or R) by either pressing a key representing “Go” or inhibiting a response by not pressing the key representing “No-Go” [15,16,19]. Test–retest reliability of mean accuracy during the familiarization session revealed a C_V_ of 4.3% and a SEM% of 0.61. Following this, participants took the Sternberg Task Test (STT). Visual stimuli (in the form of trigrams) are presented one at a time with the participant identifying them as either present or absent within sequences of at 3, 6, 9, 12, 15, or 18-s intervals. In order to prevent rehearsal, the participants were instructed to count backwards in threes and fours to a specific random number until they saw a red light appear on the computer screen [15,16]. This test measures short-term/working memory (STM/WM) involving cognitive control processes, using reaction time and accuracy [20]. Test-retest reliability of present accuracy during the familiarization session revealed C_V_’s of 3.8%, 4.9%, and 7.6% and a SEM% of 0.54, 0.69, and 1.08 for 2-letter, 4-letter, and 6-letter responses, respectively. Participants then performed the general attention Psychomotor Vigilance Task Test (PVTT). This test assesses sustained attention reaction times through responses to visual stimuli (as light) requiring participants to press a keyboard button in response to a randomly illuminating light on screen every few seconds [21,22,23]. The number of times the button was not pressed and the speed of response were measured, with sleepiness quantified as the number of lapses in attention during the test [15,16]. Test–retest reliability during the familiarization session for trials 2, 10, and 20 revealed C_V_’s of 28.6%, 44.7%, and 47.9% and a SEM% of 4.04, 6.31, and 6.77, respectively. 

### 3.4. Cambridge Brain Sciences Reasoning and Concentration Tests

Participants then performed the Cambridge Brain Sciences Reasoning and Concentration Test which was used to measure core elements of cognition (i.e., short-term memory, reasoning, attention, verbal ability). There were six assessments in this battery of cognitive tests: (1.) Rotations; (2.) Polygons; (3.) Odd One Out; (4.) Spatial Planning; (5.) Feature Match; (6.) Double Trouble. The Rotations task measures the ability to manipulate objects spatially in the mind [24]. Two grids were displayed on the computer screen with one grid rotated by a multiple of 90 degrees. The participants were required to indicate whether the grids were identical. The task duration lasted 90-s in which the participants attempted to solve as many problems as possible. The Polygons task assesses age-related disorders, particularly in measuring difficulties of successfully/incorrectly answering problems [25]. Pairs of overlapping polygons were displayed on one side of the screen and participants were required to indicate whether the other side of the screen was identical to the interlocked polygons. The Odd One Out task measures the number of correctly answered problems. Participants had 3-min to deduce rules that related to object features and identify the pattern that did not correspond to the rules. The Spatial Planning task measures planning by giving participants 3-min to reposition beads in order to configure an ascending numerical order in as few moves as possible [26]. The Feature Match task measures attention processing by giving participants 90-s to indicate whether grids displayed on the screen were identical [27]. Lastly, the Double Trouble task, a variant of the Stroop test, measures the number of correct responses in 90-s to describe the color of a word displayed at the top of the screen [28]. Test–retest reliability during the familiarization session revealed C_V_’s of 11.9%, 10.4%, 6.7%, 6.9%, and 10.1% and SEM% of 1.5%, 1.3%, 0.85%, 0.88%, and 1.29% for response inhibition (double trouble), mental rotation (rotations), visuospatial processing (polygons), deductive reasoning (odd one out), and planning (spatial planning), respectively. 

### 3.5. Light Tracking Reaction Test

A light tracking reaction performance test was administered using the NeuroTracker Pro on-site system (NeuroTracker, Montreal, Quebec, QC, Canada), running the CORE assessment (3 sessions) to measure perceptual-cognitive skill. A single CORE session included 20 trials, lasting about 6 min (8-s each), wherein 6–8 identical yellow balls were displayed on the wall (presentation phase) via 3D projector (DLP projector, Optoma Corp., New Taipei City, Taiwan), with 2–3 identified as targets (identification phase). The balls then began moving within the 3D environment at a predetermined speed (removal phase); upon stopping the participant was asked to identify which of the balls were the targets (stoppage phase), followed by immediate feedback of their accuracy (feedback phase). A typical session is based on a staircase procedure, where speed displacement is increased or decreased by ±0.05 log if the targets are correctly identified or even one is missed, respectively. The speed of the balls represents a real-world speed across the user’s field of view, measured as 68 cm/second at speed 1.0 (136 cm/second at speed 2.0, and so on). A score given at the end of the session is called the ‘speed threshold’ which represents the level at which all the targets were tracked successfully ≈ 50% of the time. The program was run on a Republic of Gamers Zephyrus GX501 gaming laptop (AsusTek Computer Inc., Taipei, Taiwan) with a Logitech G, PRO Wireless gaming mouse (Logitech, Lausanne Switzerland) gamer mouse. Participants wore BOBLOV JX-30 3D DLP-link active shutter glasses for testing. Test–retest reliability of correct targets during the familiarization session revealed a C_V_ of 6.5% and a SEM% of 0.81.

### 3.6. Side Effects Assessment

A side-effects questionnaire was used to assess whether the participants experienced and subjective side effects in response to ingesting the supplements during the experiment. Participants ranked the frequency (F) and severity (S) of experienced symptoms or side effects (i.e., dizziness, headache, tachycardia, heart skipping/palpitations, shortness of breath, nervousness, blurred vision, and any other adverse effects), if any, using; (0) none; (1) minimal 1–2/week; (2) slight 3–4/week; (3) F: occasional 5–6/week, S: moderate; (4) F: frequent 7–8/week, S: severe; or (5) F: severe ≥ 9/week, S: very severe. Test-to-test variability of this survey previously reported from our lab yielded mean C_V_’s ranging between 1.2 to 2.6 with intraclass correlations ranging between 0.59 to 0.88 for individual items on the survey [29,30]. 

### 3.7. Statistical Analysis

Data were analyzed using IBM^®^ SPSS^®^ Version 28 software (IBM Corp., Armonk, NY, USA). The sample size was determined based on the expectation of a 5% improvement with corresponding power of 0.80. Data are reported without sex as a dependent variable since participants served as their own control and there were no significant treatment × time × sex interactions observed. Missing raw data (0.01%) were extrapolated from the average of one time point immediately before and after, wherever possible. Data were analyzed using general linear models (GLM) with repeated measures univariate and multivariate analyses (MANOVA) using body weight (kg) as a covariate. Delta (∆) change values from baseline were calculated and used to determine changes from baseline. Multivariate and univariate effects are expressed through Wilks’ Lambda distributions and Greenhouse-Geisser correction tests, respectively, for time (T) and treatment × time (G × T) effects. Data were considered statistically significant when the probability of type I error (α-level) was 0.05 or less with trends being noted when p-levels ranged between *p* > 0.05 to *p* < 0.10. Fisher’s least significant difference post-hoc analysis was performed for pairwise comparisons. Dependent *t*-tests with Fisher correction post-hoc analyses were also performed at contrasts of interest. Additionally, mean changes from baseline as well as mean percentage changes from baseline with 95% CI’s and Sidak adjustment were performed. Mean changes and 95% CI’s completely above or below baseline were considered significantly different [31]. Data are presented as mean or mean change ± SD as appropriate with figures showing 95% CI’s (mean change ± SD [LL, UL]). Partial Eta squared effect sizes ($\eta_{p}^{2}$) are reported as indicators of magnitude of effect where 0.01 was considered a small effect, 0.06 was considered a medium effect, and 0.14 was considered a large effect size [32]. Additionally, Cohen’s d effect size values are reported for dependent *t*-test analysis where *d* = 0.2 were considered small, *d* = 0.5 were considered medium, and *d* = 0.8 were considered large effect sizes [32].

## 4. Results

### 4.1. Demographic Data

Table S1 presents participant demographic data. A total of 26 individuals completed the study (8 females and 18 males). Participants were 23.1 ± 5 years old, 171 ± 11 cm tall, weighed 71.1 ± 13.8 kg, had a body mass index (BMI) of 20.7 ± 3.5 kg/m^2^, had a resting heart rate of 73.2 ± 12 bpm, a systolic blood pressure of 111.6 ± 23 mmHg, and a diastolic blood pressure of 74.3 ± 6 mmHg. Significant sex differences were observed in height (*p* < 0.001), weight (*p* = 0.004), and diastolic blood pressure (*p* = 0.003) while BMI tended to be different (*p* = 0.053). For this reason, body weight was used as a covariate in GLM analyses. 

### 4.2. PEBL Cognitive Function Assessment

#### 4.2.1. Berg-Wisconsin Card Sorting Task Test

Table S2 presents results of the Berg-Wisconsin Card Sorting Task test that assesses reaction time and accuracy in measuring long thought, reasoning, learning, executive control, and attention shifting. No significant overall or univariate treatment × time interaction effects were observed from GLM analysis using weight as a covariate in correct responses, errors, perseverative errors (PEBL) or perseverative errors (PAR rules). However, mean correct responses tended to be higher in the ASI + I treatment (PLA 102.0 ± 0.6, ASI + I 103.5 ± 0.6, *p* = 0.094). Analysis of mean percentage changes from baseline with 95% CI’s revealed no significant changes over time or between treatments.

#### 4.2.2. Go/No-Go Task Test

Table S3 also presents GNG response time and accuracy results that assess sustained attention, response control to visual stimuli, and impulsiveness. No significant overall or univariate treatment × time interaction effects were observed from GLM analysis using weight as a covariate in mean accuracy or Go and No−Go task response times. However, analysis of mean changes from baseline with 95% CI’s revealed that Pre−Supplement to Pre−Game Go Task Mean Accuracy (−1.2 ± 0.6%, (−0.05, −2.34), *p* = 0.041), Round 2: Condition R response time (−4.61 ± 2.2%, (−0.25, −8.98), *p* = 0.027), and mean response time (−3.88 ± 1.9%, (−0.07, −7.69), *p* = 0.046) decreased from baseline in the PLA group with no change observed in the ASI + I treatment. This persisted in Post−Game Round 2: Condition R response times (−4.36 ± 1.9%, (−0.53, −8.19), *p* = 0.027) with a trend in mean response time (−3.03 ± 1.5%, (0.082, −6.14), *p* = 0.056) while no changes were seen in ASI + I treatment values. In No−Go tasks, there was evidence that Pre−Supplement to Pre−Game Round 2: Condition P response time tended to decrease from baseline (−5.41 ± 3.1%, (0.85, −11.7), *p* = 0.089) while Post−Game values significantly decreased from (−4.89 ± 2.3%, (−0.19, −9.59), *p* = 0.042) with ASI + I treatment with no changes with PLA treatment. Additionally, Post−Game mean response time decreased from baseline in the PLA treatment (4.05 ± 1.9%, (0.15, 7.95), *p* = 0.042). However, no significant differences were observed between treatments. 

**Figure 3:**
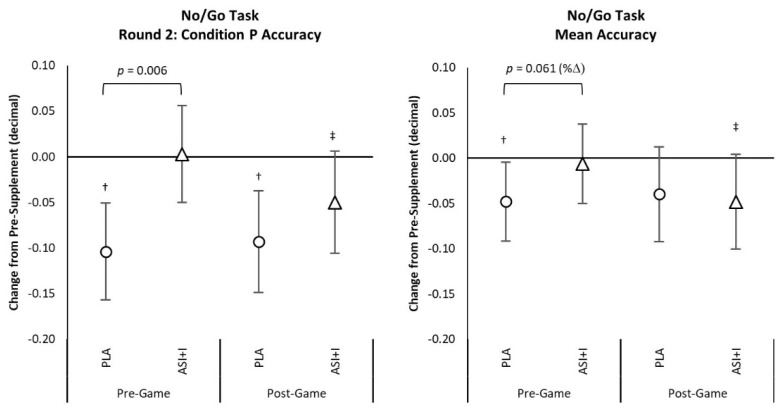
Mean changes from baseline in selected No/Go Task data. † represents *p* < 0.05 change from baseline values. ‡ represents *p* > 0.05 to *p* < 0.10 difference from baseline values.

Table S3 also presents GNG accuracy results expressed as a decimal percentage. No significant overall or univariate treatment × time interaction effects were observed from GLM analysis using weight as a covariate in percent accuracy parameters. Analysis of mean changes from baseline with 95% CIs found that Pre−Game No−Go Task Round 2: Condition *p* mean accuracy was significantly higher in the ASI + I treatment compared to PLA (22.63 ± 8.5%, (5.6, 39.8), *p* = 0.01) and tended to be higher in mean accuracy (15.5 ± 8.1%, (−0.7, 0.6), *p* = 0.061). There was also some evidence that No−Go Round 2: Condition *p* values (−17.4 ± 6.0%, (−5.4, −19.4), *p* = 0.006) and mean accuracy (−11.5 ± 5.7%. (−0.05, −22.9), *p* = 0.049) were significantly decreased from Pre−Supplement to Pre−Game in the PLA treatment with no changes in the ASI + I treatment (see Figure 3). 

#### 4.2.3. Sternberg Task Test

**Figure 4:**
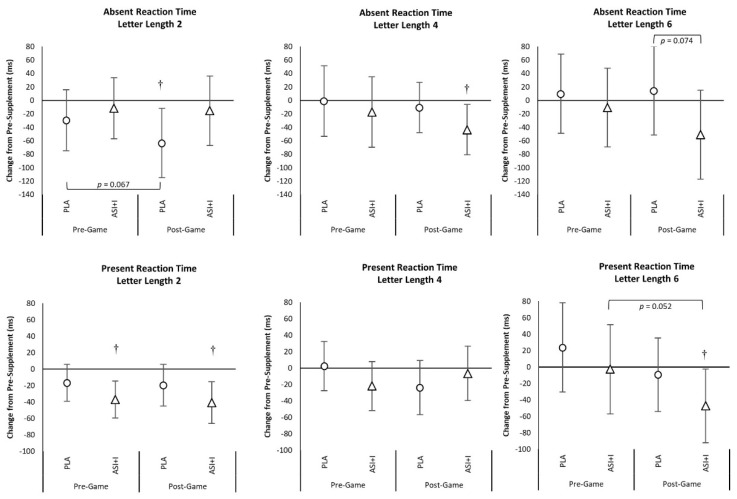
Mean changes with 95% Confidence Intervals in Absent and Present Reaction Times. † represents *p* < 0.05 difference from baseline.

Table S4 presents Sternberg Task Test reaction time results as well as data expressed as percent accuracy. No significant overall or univariate treatment × time interaction effects were observed from GLM analysis using weight as a covariate in reaction time data. However, analysis of changes from baseline with 95% CI’s (Figure 4) revealed that Pre-Game Present Reaction Time Letter Length 2, Post-Game Absent Reaction Time Letter Length 4, Pre-Game and Post-Game Present Reaction Time Letter Length 2, and Post-Game Present Reaction Time Letter Length 6 values significantly decreased from baseline in the ASI + I treatment while only Post-Game Absent Reaction Time Letter Length 2 values decreased from baseline with PLA treatment. Additionally, Post-Game Present Reaction Time Letter Length 6 values tended to be faster between treatments with ASI + I treatment (−62.3 ± 34.1 ms, [6.2, −130.7], *p* = 0.074). Post-hoc dependent *t*-test analysis found significant differences between treatments in Post-Game 4 Letter Absent Reaction time (*p* = 0.05, *d* =0.31), 6 Letter Present Reaction Time (*p* < 0.01, *d* = 0.41), 6 Letter Absent Reaction Time (*p* = 0.01, *d* = 0.36), Mean Present Reaction Time (*p* = 0.02, *d* = 0.21), and Mean Absent Reaction Time (*p* = 0.03, *d* = 0.24). Significant treatment × time interactions were observed in Letter Length 2 Present Accuracy (*p* = 0.038) and Mean Present Accuracy (*p* = 0.025) with a trend and small-medium effect size observed in Letter Length 6 Present Accuracy (*p* = 0.102, $\eta_{p}^{2}$ = 0.046). Dependent *t*-test analysis also revealed significant differences between treatments in Post-Game 2 Letter Present Accuracy (*p* = 0.02, *d* = 0.55) and mean Present Accuracy (*p* = 0.01, *d* = 0.46).

#### 4.2.4. Psychomotor Vigilance Task Test

Table S5 presents Psychomotor Vigilance Task Test Data. No significant overall or univariate treatment x time effects were observed. Likewise, analysis of mean changes from baseline with 95% CI’s revealed no changes from baseline or between treatments in Trial 2, Trial 10, Trial 20, or mean Reaction Time responses. 

### 4.3. Cambridge Brain Sciences Reasoning and Concentration Tests

Table S6 presents the Cambridge Brain Reasoning and Concentration Test results. No significant overall or univariate treatment × time effects were observed. Analysis of mean changes from baseline with 95% CI’s revealed that response inhibition (i.e., the ability to concentrate on relevant information to make a correct response) increased from baseline in PLA and ASI + I treatments with no differences observed between treatments. There was also evidence that Spatial Planning that assesses ability to act without forethought and sequence behavior in an orderly fashion significantly increased from baseline with ASI + I treatment (Pre-Game 4.57 ± 2.2%, (−0.2, 8.9), *p* = 0.041; Post-Game 6.43 ± 2.3%, (1.8, 11.1), *p* = 0.008) with no significant differences observed between treatments. 

### 4.4. Light Tracking Reaction Test

Table S7 presents light reaction test results. No significant overall or univariate treatment × time effects were observed between treatments. Analysis of changes from baseline with 95% CIs revealed that the ratio of correct targets hit during the session and start speed threshold (i.e., level at which targets can be tracked successfully 50% of the time) increased over time in both treatments while score values (i.e., a measure of distributed or divided attention, selective attention, sustained attention, and attention stamina) decreased from baseline with no significant differences observed between treatments. Total test times did not change over time in either treatment. 

### 4.5. Safety Assessment

Table S8a,b show the frequency and severity of side effects evaluated in this study, respectively. Chi squared analysis revealed that no significant differences were observed between treatments in perceptions of the frequency or severity of dizziness, headache, tachycardia, heart palpitations, dyspnea, nervousness, blurred vision, or other complaints. Side effects reported were infrequent and of little severity when reported. 

## 5. Discussion

Playing e-sports requires neurocognitive expertise to react quickly to moving objects, and advanced thought and reasoning to navigate through obstacles in order to achieve game objectives [1]. Improving the ability to focus, quickly react to visual and auditory stimuli, and/or sustain attention during long games and/or a series of games would seemingly be beneficial to gamers. For this reason, there has been interest in determining if nutritional nootropics may provide ergogenic benefit to gamers. This study examined whether inositol stabilized arginine silicate, a substance shown to increase arginine and NO levels, and whether additional inositol affects cognitive function, reaction time, and/or memory in experienced e-sport gamers following a 1-h gaming challenge. Given evidence in prior studies [4,11,12], we hypothesized that ingestion of ASI + I prior to a gaming challenge would affect cognitive function. Results of this study provide evidence that acute ASI + I ingestion improved primary outcomes of reaction time and working memory among e-sport gamers in several cognitive function related tests with no reported stimulant-related side effects. The following provides additional discussion regarding these findings. 

Nitric oxide promotes vasodilation thereby increasing blood flow to tissues, including the brain [5,6,7]. Inositol stabilized arginine silicate has been reported to increase arginine and nitric oxide levels in the blood [4]. Additionally, inositol has been reported to enhance neurotransmission and memory [8,9,10]. Prior research from Kalman et al. [11] indicated that 3-days of ASI supplementation (1500 mg/d) decreased Trail Making Test (TMT) part B performance times by 35% compared to placebo while 14-days of supplementation improved performance on this test by 28% [11]. The trail making test (TMT) asks participants to draw lines between consecutive circles displayed randomly on a page (TMT-A uses all numbers while the more complex TMT-B uses alternating numbers and letters) [33,34]. The TMT-B involves working memory, visual-spatial skills, as well as motor and executive functions [33,35]. These findings suggest that ASI supplementation enhanced visual attention, search speed, processing speed, mental flexibility, and executive functioning [36].

Similarly, Tartar et al. [12] reported that 1-day and 7-days of 1600 mg of inositol-enhanced bonded arginine silicate supplementation increased perceptions of energy and decreased TMT-B test errors compared to placebo. Additionally, fatigue, TMT-B time, and TMT-A score significantly improved from baseline while post-game TMT-A time improved with ASI + I supplementation. They also reported that post-game Stroop color word test errors decreased with ASI + I. The Stroop test involves asking participants to speak aloud the color in which a word is displayed (color naming) and not read the word which spells out a different color (word reading). It has been used to assess the relationship between response inhibition and working memory [37,38]. More specifically, word-reading reflects speed of visual search, color-naming reflects working memory and speed of visual search, and color-word identification reflects working memory, conflict monitoring, and speed of visual search [39]. The researchers concluded that ASI ingestion may serve as an effective ergogenic aid for esports athletes looking to improve accuracy, decision making, and/or reaction time during gaming [12].

This study examined whether acute ASI + I ingestion prior to playing video games affects cognitive and/or executive function and/or reaction time in experienced gamers, and whether ASI + I supplementation affected different aspects of cognitive function, focus, attention, and/or memory in gamers than had previously been studied. This was accomplished by having participants undergo a battery of cognitive function tests that assess different types of cognitive function prior to and following 15-min of ingestion of ASI + I as well as after 1-h of playing video games. This included having participants take the Berg-Wisconsin Card Sorting Test which assesses reaction time and accuracy in measuring long thought, reasoning, learning, executive control, attention shifting and impulsiveness [17,18] and is often used to identify impairment due to brain disorders or damage [40]; the Go/No-Go test which assesses sustained attention and response control through reaction time and accuracy of responding to visual stimuli [15,16,19]; the Sternberg Task Test which measures short-term/working memory involving cognitive control processes, using reaction time and accuracy [20,41,42,43].; the Psychomotor Vigilance Task Test which assesses sustained attention reaction times through responses to visual stimuli [21,22,23]; the Cambridge Brain Sciences Reasoning and Concentration Test which measures core elements of cognition (i.e., short-term memory, reasoning, attention, verbal ability) [24]; and a Light Reaction Test which assessed the speed in recognizing and tagging targets. 

Results of the present study provide evidence that acute ASI + I ingestion improved reaction times and working memory in several tests. In this regard, there was evidence that ASI + I improved GNG reaction time and accuracy as well as speed and reaction time of the Sternberg Test in multiple letter lengths of increasing difficulty and on average versus the placebo group. These findings are consistent with prior reports showing that ASI and/or ASI + I improved cognitive function as assessed by the Stroop color word test and Trail Making Test that also target working memory [11,12]. There was also evidence that Spatial Planning that assesses ability to act without forethought and sequence behavior in an orderly fashion was improved with ASI + I. Conversely, ASI + I had no significant effects on the Berg-Wisconsin Card Sorting test, the Psychomotor Vigilance Task Test, or the Light Tracking Reaction Test. These findings suggest the primary effects of ASI + I ingestion involve enhancing short-term and working memory, reaction time, reasoning, and concentration in experienced gamers and that gaming interventions may serve as an effective way to assess neurocognition [1]. Results support contentions that ingesting nutrients like ASI + I that increase blood arginine and nitric oxide [4] may enhance cognitive function and memory [5,6] possibly by improving blood flow and oxygen availability [7] and/or enhancing neurotransmission [8,9,10]. However, more research is needed to determine mechanisms of action. 

Results contrast findings from Thomas et al. [3] who evaluated the effects of energy drink consumption on cognitive and physical performance in nine elite League of Legends gamers. In a similarly designed study, participants underwent a cognitive and physical test battery prior to consuming a placebo or AI Reload (AI Fuels Llc. Santa Monica, CA, USA) containing 150 mg of Caffeine (1.9 ± 0.3 mg/kg), L-theanine, Phosphatidylserine, Choline [from alpha-GPC], and Nicotinamide Adenine Dinucleotide [reduced form NADH]). After 30 min, participants played three League of Legend games followed by performing the cognitive and physical test battery. The researchers found no significant differences between the placebo and AI Reload ingestion on mental or physical improvement in performance during this gaming challenge, including on the Go/No-Go test. While additional research is needed, it is interesting to note that ingesting nutrients that have been reported to promote increases in nitric oxide appeared to have a greater effect on cognition than consuming an energy drink with a moderate dose of caffeine and other nootropic nutrients. 

## 6. Conclusions

In conclusion, results of the present investigation provide additional evidence that acute supplementation of ASI + I can affect cognitive function and memory in competitive gamers. The strength of this study was that it evaluated the acute effects of ingesting ASI + I on a spectrum of cognitive function tests in experienced gamers. This helped further identify the potential ergogenic benefits in this population. Limitations include the number of females studied and the amount of time it took to take the test battery, which could have influenced test fatigue and results of tests performed toward the end of the test battery. Additional research should further examine the effects of acute and chronic ASI + I ingestion on cognitive function, executive function, and memory in gamers. Moreover, research should examine how acute and chronic ASI + I may affect cognitive function over time in healthy younger and older populations.

## Data Availability

Data and/or statistical analyses are available upon request on a case-by-case basis for non-commercial scientific inquiry and/or educational use as long as IRB restrictions and research agreement terms are not violated.

## Supplementary Materials

The following are available online at https://www.mdpi.com/article/10.3390/nu13113758/s1, Table S1: Participant demographic data, Table S2: Berg-Wisconsin Card Sorting Task Test results, Table S3: Go/No-Go Task Test response time and accuracy results, Table S4: Sternberg Task Test reaction time results, Table S5: Psychomotor Vigilance Task Test results, Table S6: Cambridge Brain Reasoning and Concentration Test results, Table S7: Light Tracking Reaction Test results, Table S8: Side Effects Analysis.
